# Supplementary material for: Characterizing cell-type spatial relationships across length scales in spatially resolved omics data
Source: Nat Commun. 2025 Jan 3;16:350. doi: 10.1038/s41467-024-55700-1 (PMC11699133; doi:10.1038/s41467-024-55700-1)
Supplement: Supplementary file 3 — Supplementary Software 1 [file 41467_2024_55700_MOESM3_ESM.zip › CRAWDAD/plots/spleen/3_spleen_pkhl_grid_tissue.pdf]

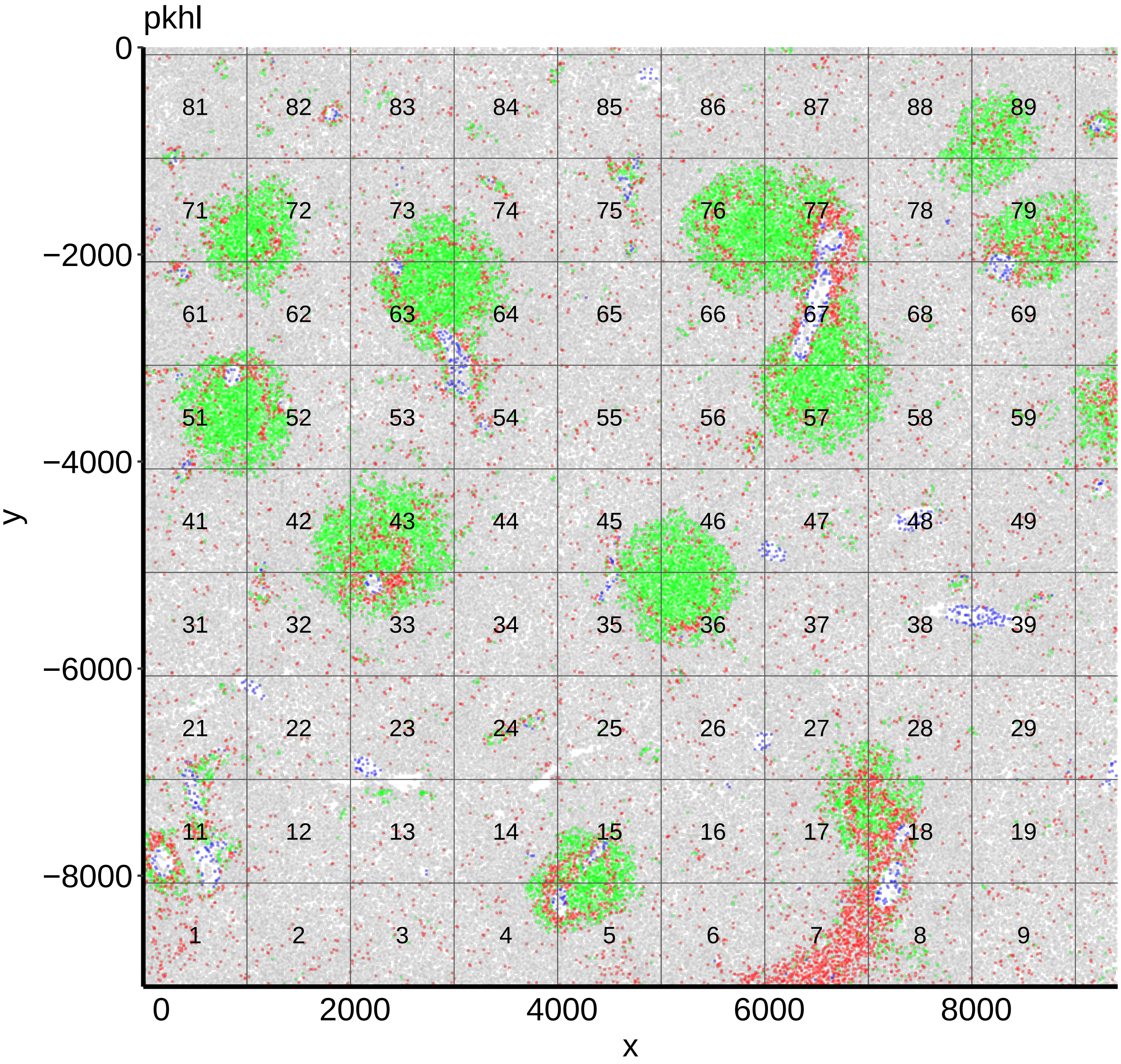
pkhl
0
−2000
−4000
−6000
−8000
y
0
2000
4000
6000
8000
x
81 82 83 84 85 86 87 88 89
71 72 73 74 75 76 77 78 79
61 62 63 64 65 66 67 68 69
51 52 53 54 55 56 57 58 59
41 42 43 44 45 46 47 48 49
31 32 33 34 35 36 37 38 39
21 22 23 24 25 26 27 28 29
11 12 13 14 15 16 17 18 19
1 2 3 4 5 6 7 8 9
